# *Salmonella enterica* serovar Typhimurium Δ*msbB* Triggers Exacerbated Inflammation in Nod2 Deficient Mice

**DOI:** 10.1371/journal.pone.0113645

**Published:** 2014-11-25

**Authors:** Anne-Kathrin Claes, Natalie Steck, Dorothee Schultz, Ulrich Zähringer, Simone Lipinski, Philip Rosenstiel, Kaoru Geddes, Dana J. Philpott, Holger Heine, Guntram A. Grassl

**Affiliations:** 1 Institute for Experimental Medicine, University of Kiel, Kiel, Germany; 2 Division Models of Inflammation, Research Center Borstel, Borstel, Germany; 3 Division Immunochemistry, Research Center Borstel, Borstel, Germany; 4 Institute of Clinical Molecular Biology; University of Kiel, Kiel, Germany; 5 Department of Immunology, University of Toronto, Toronto, Ontario, Canada; 6 Division of Innate Immunity, Research Center Borstel, Member of the Airway Research Center North (ARCN) of the German Center for Lung Research (DZL), Borstel, Germany; University of Osnabrueck, Germany

## Abstract

The intracellular pathogen *Salmonella enterica* serovar Typhimurium causes intestinal inflammation characterized by edema, neutrophil influx and increased pro-inflammatory cytokine expression. A major bacterial factor inducing pro-inflammatory host responses is lipopolysaccharide (LPS). *S.* Typhimurium Δ*msbB* possesses a modified lipid A, has reduced virulence in mice, and is being considered as a potential anti-cancer vaccine strain. The lack of a late myristoyl transferase, encoded by MsbB leads to attenuated TLR4 stimulation. However, whether other host receptor pathways are also altered remains unclear. Nod1 and Nod2 are cytosolic pattern recognition receptors recognizing bacterial peptidoglycan. They play important roles in the host's immune response to enteric pathogens and in immune homeostasis. Here, we investigated how deletion of *msbB* affects *Salmonella's* interaction with Nod1 and Nod2. *S.* Typhimurium Δ *msbB*-induced inflammation was significantly exacerbated in *Nod2* ^−/−^ mice compared to C57Bl/6 mice. In addition, *S.* Typhimurium Δ*msbB* maintained robust intestinal colonization in *Nod2* ^−/−^ mice from day 2 to day 7 p.i., whereas colonization levels significantly decreased in C57Bl/6 mice during this time. Similarly, infection of *Nod1* ^−/−^ and *Nod1/Nod2* double-knockout mice revealed that both Nod1 and Nod2 play a protective role in *S.* Typhimurium Δ*msbB*-induced colitis. To elucidate why *S.* Typhimurium Δ*msbB*, but not wild-type *S.* Typhimurium, induced an exacerbated inflammatory response in *Nod2* ^−/−^ mice, we used HEK293 cells which were transiently transfected with pathogen recognition receptors. Stimulation of TLR2-transfected cells with *S.* Typhimurium Δ*msbB* resulted in increased IL-8 production compared to wild-type *S.* Typhimurium. Our results indicate that *S.* Typhimurium Δ*msbB* triggers exacerbated colitis in the absence of Nod1 and/or Nod2, which is likely due to increased TLR2 stimulation. How bacteria with “genetically detoxified” LPS stimulate various innate responses has important implications for the development of safe and effective bacterial vaccines and adjuvants.

## Introduction

*Salmonella enterica* sv. Typhimurium is a Gram-negative food-borne pathogen causing enterocolitis and it is a major global health burden. *Salmonella* is recognized by the host through pattern recognition receptors (PRRs) such as toll-like receptors (TLRs) and nucleotide-binding oligomerization domain (Nod)-like receptors (NLRs). Several PRRs detect bacterial cell wall components, e.g. TLR4 recognizes bacterial lipopolysaccharide (LPS), TLR2 recognizes lipoproteins, and Nod1 and Nod2 both recognize peptidoglycan degradation products [1]. Nod2 recognizes muramyl dipeptide (MDP) and the ligand for Nod1 is meso-diaminopimelic acid (ieDAP) and they are both important factors for host defense against intracellular pathogens [2] [3] [4]. Activation of PRRs is a crucial step for the host's immune system to mount an appropriate inflammatory response against bacterial infections.

Previous studies have shown that the lack of either Nod1 or Nod2 had no effect on the extent of *Salmonella*-triggered intestinal inflammation whereas *Salmonella* caused reduced colitis in a Nod1/Nod2 double-knockout (DKO) or in a *Rip2^−/−^* mouse [5], which is the signaling molecule downstream of Nod1 and Nod2. Aberrant triggering of Nod2 can lead to the development of inflammatory bowel diseases such as ulcerative colitis or Crohn's disease (CD). Non-functional mutations of Nod2 are major risk factors for CD [6] [7] [8]. However, how the lack of Nod2-signaling leads to increased chronic inflammation remains unclear.

Recent studies have shown that crosstalk between TLRs and Nod2 plays an important role in the regulation of innate immune signaling. In particular, synergistic crosstalk of Nod2 with TLR2 and/or TLR4 enhances cytokine production and strengthens intestinal barrier function [9] [10] [11]. Interestingly, other studies have revealed an antagonistic role of Nod2 in TLR signaling. Richardson and colleagues identified Nod2 as a negative regulator of TLR4 in necrotising enterocolitis (NEC) as prestimulation with MDP led to milder LPS-induced NEC in newborn mice [12]. *In vitro* experiments showed that the dampening effect of Nod2 on TLR4 signaling requires the CARD (caspase activation and recruitment domain) and LRR (leucin-rich repeat) domains of Nod2 [13]. Hedl *et al.* demonstrated that acute stimulation of Nod2 leads to a synergistic effect on TLR signaling while chronic stimulation results in down-regulation of TLR responses [14]. These studies suggest that the outcome of the Nod-TLR crosstalk depends on the context of stimulation.

LPS is an important virulence determinant for *S.* Typhimurium and it consists of lipid A, the core oligosaccharide and the O antigen [15]. Several modifications including the extent of acylation of lipid A influence its ability to activate TLR4. The *Salmonella* enzyme MsbB modifies LPS by adding a myristic acid residue onto lipid A resulting in a hexa-acylated LPS. In addition, activity of the acyl transferase PagP adds a palmitic acid onto the complete lipid A making hepta-acylated lipid A. As a result, wild-type *Salmonella* LPS contains a mixture of hexa- and hepta-acylated lipid A while the Δ*msbB* mutant lacks one acyl chain, therefore having a mixture of penta- and hexa-acylated lipid A [16]. The Δ*msbB* mutant LPS is an agonist for TLR4, however it induces weaker proinflammatory signalling than wildtype LPS. The reason for this is it contains both hexa-acylated lipidA (which is a strong TLR4 stimulator) and penta-acylated lipid A which does not stimulate TLR4) whereas wildtype LPS contains hexa-acylated lipidA and hepta-acylated lipid A (both strong TLR4 stimulators). The pro-inflammatory ability of Δ*msbB* mutant LPS and the influence of the number of acyl chains on proinflammatory signaling has thoroughly been demonstrated by Matsuura and colleagues [17]. Changes in LPS composition can also influence the overall composition of the bacterial cell wall and/or the accessibility to other cell wall constituents such as lipoproteins or peptidoglycans.

Here, we investigated how differences in LPS composition affect *Salmonella* triggered colitis using the streptomycin pretreated mouse model [18] [19]. We demonstrate that *S.* Typhimurium Δ*msbB* infection triggered exacerbated inflammation in *Nod1^−/−^*, *Nod2^−/−^* and DKO mice compared to C57BL/6 mice. In addition, using *in vitro* transfection of TLRs or NLRs into HEK293 cells we demonstrate that *S.* Typhimurium Δ*msbB* displays no differences compared to wild-type *S.* Typhimurium with regard to Nod2 stimulation. In contrast, *S.* Typhimurium Δ*msbB* showed strongly increased TLR2 mediated pro-inflammatory cytokine production. Our results indicate that Nod1 and Nod2 function as modulators of intestinal inflammation by inhibition of TLR2 signaling and thereby prevent excessive triggering of TLR-dependent inflammation.

## Materials and Methods

### Bacterial strains

*S.* Typhimurium C5 wild-type [20] and *S.* Typhimurium C5 Δ*msbB* [16] were grown in 2 ml LB-broth containing 100 µg/ml streptomycin or 50 µg/ml kanamycin while shaking at 37°C over night.

### Mouse experiments

C57Bl/6J and B6.129S1-Nod2^tm1Flv^/J (*Nod2* ^−/−^) [21] mice were purchased from the Jackson Laboratory (Bar Harbor, Maine, USA). The *Nod2* ^−/−^ line was back-crossed to C57Bl/6J for at least 10 generations in the animal facility of the University of Kiel, Germany. Mice were treated with 20 mg streptomycin by oral gavage 24 h prior to infection with 3×10^6^ *S.* Typhimurium wild-type or 3×10^7^ *S.* Typhimurium Δ*msbB* by oral gavage and sacrificed at indicated time points. Tissue samples were collected at various time points for further investigations. These animal experiments were performed in the mouse facility of the Research Center Borstel (FZB), Germany. For some experiments, C57Bl/6, *Nod1^−/−^*, *Nod2^−/−^* and *Nod1^−/−^*/*Nod2^−/−^* mice were bred and animal experiments were performed in the mouse facility of the University of Toronto, Canada and were approved by the Animal Ethics Committee of the University of Toronto. Mice were housed under specific pathogen-free conditions in individual ventilated cages. Food and water were provided ad libitum.

### Ethics statement

All experiments were conducted consistent with the ethical requirements of the Animal Care Committee of the Ministry of Energy, Agriculture, the Environment and Rural Areas of Schleswig-Holstein, Germany and in direct accordance with the German Animal Protection Law. The protocols were approved by the Ministry of Energy, Agriculture, the Environment and Rural Areas of Schleswig-Holstein, Germany (Protocol: V312-72241.123-3(65-5/09).

### Bacterial tissue colonization

Tissue samples of the cecum, colon, ileum, spleen, liver and mesenteric lymph nodes (MLN) were homogenized in 1 ml sterile phosphate-buffered saline (PBS) using a TissueLyser II (Qiagen, Hilden, Germany). Serial dilution of the homogenate were performed and plated on LB agar plates containing 100 µg/ml streptomycin or 50 µg/ml kanamycin.

### Histology

Tissue samples of the cecum were fixed in formalin, embedded in paraffin and 5 µm sections were stained with Hematoxylin & Eosin (H&E). Inflammation of the cecum was evaluated using a pathology scoring system as previously described [22].

### Immunohistochemistry

Formalin-fixed paraffin embedded sections (5 µm) were deparaffinized and rehydrated. After antigen retrieval with citrate buffer, immunostaining was performed using antibodies against CD3 (Abcam, Cambridge, UK), CD68 (Abcam), E-cadherin (Abcam) and myeloperoxidase (MPO; Thermo Fisher Scientific, Waltham, USA) followed by fluorescently-labeled secondary antibodies (Life Technologies, Carlsbad, USA). Analysis was performed using an Axio Observer.Z1 microscope (Zeiss, Wetzlar, Germany). For each mouse, the number of stained cells was counted in six randomly selected high power fields (HPF, 630× magnification) containing the cecal submucosa and mucosa (for MPO+ cells) and containing the cecal mucosa (for CD68+ cells).

### Quantitative Real-Time PCR

RNA from the cecum was isolated using High Pure RNA Tissue Kit (Roche, Mannheim, Germany) and reverse transcribed using Transcriptor HighFidelity cDNA Synthesis Kit (Roche). Expression of mRNA was quantified by real-time PCR using LightCycler480 SYBR Green I Master (Roche). Sequences of forward and reverse primers are listed in Table 1. PCR products were amplified with the following program on a LightCycler480 (Roche): 95°C for 10 minutes followed by 39 cycles of 94°C for 15 seconds and 60°C for 30 seconds. Glyceraldehydephosphate-dehydrogenase levels (GAPDH) were used for normalization. The fold difference in expression was calculated as 2^−^^ΔΔC(t)^.

**Table 1:** Primer sequences used for quantitative real-time PCR.

Gene	Sequence	
	*forward*	*reverse*
*mcp1*	ATTGGGATCATCTTGCTGGT	CCTGCTGTTCACAGTTGCC
*tnfα*	AGGGTCTGGGCCATAGAACT	CCACCACGCTCTTCTGTCTAC
*gapdh*	ATTGTCAGCAATGCATCCTG	ATGGACTGTGGTCATGAGCC
*tlr2*	TGTCTCCACAAGCGGGACTT	TTCGATGGAATCGATGATGTTG
*tlr4*	TGACAGGAAACCCTATCCAGAGTT	TCTCCACAGCCACCAGATTCT

### Stimulation of transiently transfected HEK293 cells

HEK293 cells were incubated for 24 h with 100 ng plasmid coding for either human Nod2, human TLR2 or human TLR4 (including CD14 and MD2) and lipofectamine 2000 (Life Technologies) according to the manufacturer's instruction. Next, transiently transfected cells were stimulated with the respective agents. DMEM medium served as negative control and TNF-α, MDP, P3CSK4 and LPS as corresponding positive controls, while purified LPS of either *S.* Typhimurium wild-type or *S.* Typhimurium Δ*msbB* as well as heat-killed bacteria were used for investigation. To examine the stimulation of PRRs, IL-8 production was measured using human IL-8 CytoSet ELISA (Life Technologies) according to the manufacturer's instruction.

### Statistical analysis

Statistical analyses were performed using GraphPad Prism 5 (GraphPad, San Diego, USA). Kolmogorov-Smirnov test was used to analyze normal distribution. Significance of normally distributed data was analyzed using either Student's *t* test or one-way ANOVA with Bonferroni's or Tukey's multiple comparison post-test as indicated. Not normally distributed data were analyzed by ANOVA with appropriate post-test after logarithmic transformation. Significant differences were indicated as follows: *: *p*<0.05; **: *p*<0.01; ***: *p*<0.001.

## Results

### Acute infection with *S.* Typhimurium Δ*msbB* leads to exacerbated cecal inflammation in *Nod2* ^−/−^ mice

**Figure 1:**
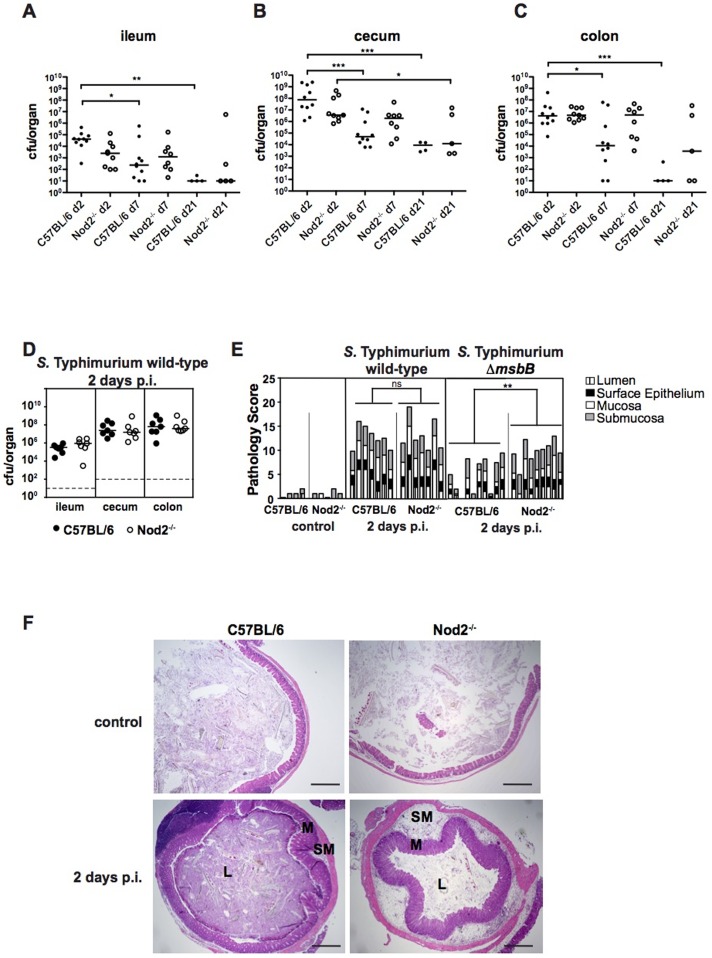
Impaired bacterial clearance and exacerbated inflammation in *Nod2^−/−^* mice. Streptomycin-pretreated C57Bl/6 and *Nod2^−/−^* mice were orally infected with wild-type *S.* Typhimurium or the Δ*msbB* mutant for the indicated times. *S.* Typhimurium Δ*msbB* colonization of the (A) ileum, (B) cecum and (C) colon. The dashed line indicates the limit of detection. cfu: colony forming units. Statistical analysis: one-way ANOVA with Tukey's multiple comparison post-test after logarithmic transformation. (D) *S.* Typhimurium wild-type colonization 2 days post infection. (E) Pathology scores of *S.* Typhimurium infected ceca and mock-infected controls at day 2 p.i., Statistical analysis: Student's *t* test. (F) H&E staining of *S.* Typhimurium Δ*msbB* infected cecal sections and mock-infected controls at day 2 p.i. Original magnification: 40×; scale bars = 500 µm; L = lumen, M = mucosa, SM = submucosa. * *p*<0.05; ** *p*<0.01; *** *p*<0.001; ns: not significant.

TLR4-dependent signaling plays an important role during *Salmonella* triggered inflammation and LPS from *S.* Typhimurium Δ*msbB* is known to have diminished TLR4-activating properties [17]. Due to a potential crosstalk between TLR4 and Nod2, we compared intestinal inflammation caused by wild-type and Δ*msbB Salmonella* in both C57Bl/6 and *Nod2^−/−^* mice. Streptomycin-pretreated C57Bl/6 and *Nod2^−/−^* mice were infected with wild-type *S.* Typhimurium or the Δ*msbB* mutant and sacrificed at indicated time points. At two days post-infection (p.i.), colonization of the intestine (Figure 1A–D) and systemic organs (not shown) of C57Bl/6 and *Nod2^−/−^* mice was similar for both wild-type and Δ*msbB S.* Typhimurium. However, in C57Bl/6 mice, bacterial loads in the ileum, cecum, and colon significantly decreased between days 2 and 7 p.i., while in *Nod2^−/−^* mice, bacterial numbers remained high. These data indicate that Nod2 is involved in bacterial clearance.

Infection of C57Bl/6 and *Nod2^−/−^* mice with wild-type *S.* Typhimurium showed no difference in intestinal colonization or inflammation, as assessed by pathological scoring of H&E stained tissue sections (Figure 1D–E). Our observations were obtained using wild-type *S.* Typhimurium C5 strain and corroborate previously published results with another wild-type *S.* Typhimurium strain (SL1344) (Figure S1A and [5]) and suggest Nod2 does not play a role in wild-type *S.* Typhimurium triggered intestinal inflammation. H&E staining also revealed that infection of C57Bl/6 mice with the *S.* Typhimurium Δ*msbB* mutant results in less cecal inflammation than infection with the wild-type strain (Figure 1E). This is thought to be due to the inability of the “genetically detoxified” Δ*msbB* LPS to efficiently stimulate TLR4 signaling.

Interestingly however, at day 2 p.i., *S.* Typhimurium Δ*msbB*-triggered inflammation was significantly exacerbated in *Nod2^−/−^* mice compared to C57Bl/6 mice (Figure 1E–F). This was not due to differences in the levels of bacterial colonization (Figure 1A–C). At 2 days p.i. histopathological analysis of the cecum of *S.* Typhimurium Δ*msbB*-infected *Nod2^−/−^* mice showed more severe submucosal edema, an advanced destruction of the crypt structure, more apoptotic epithelial cells and neutrophils in the lumen as well as greater infiltration of immune cells into the cecal mucosa (Figure 1E, F). On day 7 p.i., *S.* Typhimurium Δ*msbB*-induced inflammation was more pronounced than on day 2 but similar in both C57Bl/6 and *Nod2^−/−^* mice (Figure S1B). Therefore, Nod2 contributes to clearance and early intestinal inflammation triggered by the *S.* Typhimurium Δ*msbB* mutant.

### Nod2 delays early immune cell infiltration in *S.* Typhimurium Δ*msbB*-induced inflammation

**Figure 2:**
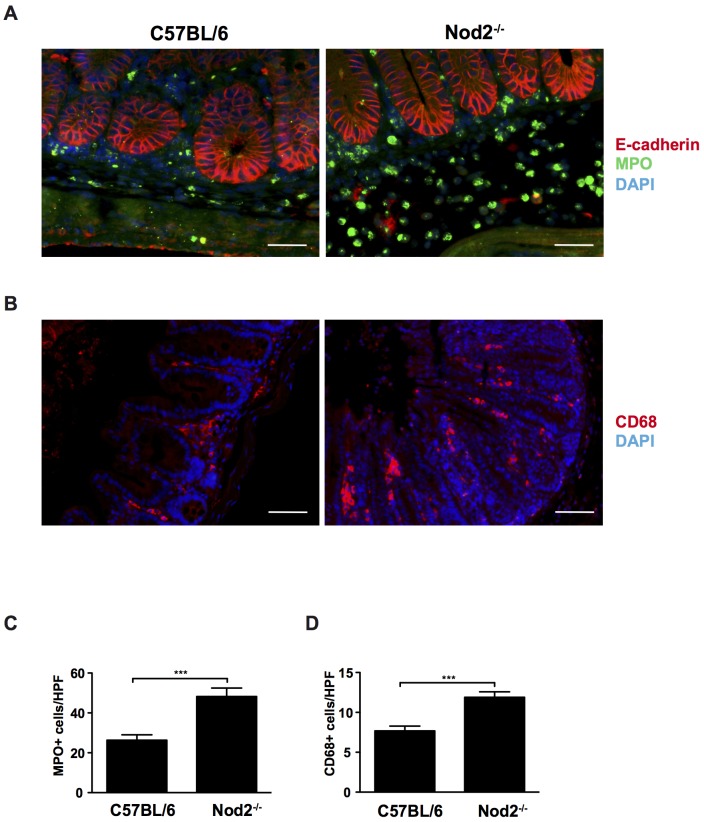
Increased early influx of neutrophils and macrophages in *Nod2^−/−^* mice. Streptomycin-pretreated C57Bl/6 and *Nod2* ^−/−^ mice were orally infected *with S.* Typhimurium Δ*msbB* for 2 days and cecum sections were stained to visualize neutrophils and macrophages, respectively. (A) Cecum sections were stained for DAPI (blue), E-cadherin (epithelial cells, red) and MPO (neutrophils, green). (B) Cecum sections were stained for DAPI (blue) and CD68 (macrophages, red). Original magnification: 400×, scale bars = 50 µm. (C) Quantification of MPO+ and (D) CD68+ cells. HPF = high power field. Statistical analysis: Student's *t* test. *** *p*<0.001.

We observed an early influx of immune cells in *Nod2^−/−^* mice upon *S.* Typhimurium Δ*msbB* infection in H&E stained cecum sections. To identify which cells were recruited to the site of infection, tissue sections were stained for myeloperoxidase (MPO) and CD68 to analyze the influx of neutrophils and macrophages, respectively. Immunostainings revealed that on day 2 p.i., the cecal tissue and lumen of *S.* Typhimurium Δ*msbB*-infected *Nod2^−/−^* mice was more highly infiltrated by neutrophils than in C57Bl/6 mice (Figure 2A,C). Similarly, Δ*msbB*-infected *Nod2^−/−^* mice had more CD68-positive macrophages in the mucosa than C57Bl/6 mice (Figure 2B,D). In contrast, there were no differences in CD3^+^ T cells in the ceca of Δ*msbB*-infected C57Bl/6 and *Nod2^−/−^* mice (not shown).

**Figure 3:**
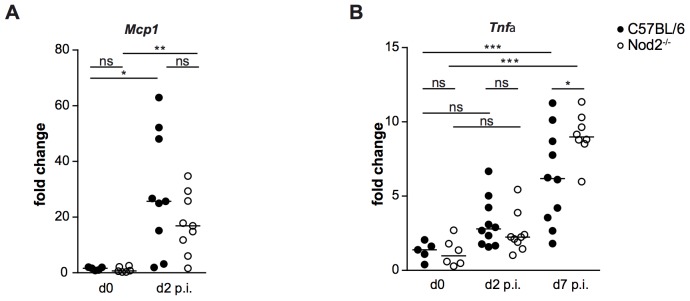
*S.* Typhimurium Δ*msbB* infection induces pro-inflammatory gene expression in C57Bl/6 and *Nod2^−/−^* mice. Gene expression of *mcp1* and *tnfα*, were measured by quantitative real-time PCR. Data were normalized to *gapdh* expression levels; Statistical analysis: 1way ANOVA with Tukey's multiple comparison post-test. * *p*<0.05; ** *p*<0.01; *** *p*<0.001; ns: not significant.

To further assess the role of Nod2 in *S.* Typhimurium Δ*msbB*-induced inflammation, pro-inflammatory cytokines MCP-1 and TNF-α were analyzed by quantitative real-time PCR (RT-PCR) in cecal tissue. At day 2 p.i., *S.* Typhimurium Δ*msbB* induced elevated levels of MCP-1 and TNF-α in *Nod2^−/−^* mice compared to C57Bl/6 (Figure 3). At this time point significant differences between C57Bl/6 and *Nod2^−/−^* mice were not detected, however, at day 7 p.i. *Nod2^−/−^* mice had significantly higher TNF-α levels than C57Bl/6 mice.

### *S.* Typhimurium Δ*msbB* LPS has increased TLR2 activation activity

**Figure 4:**
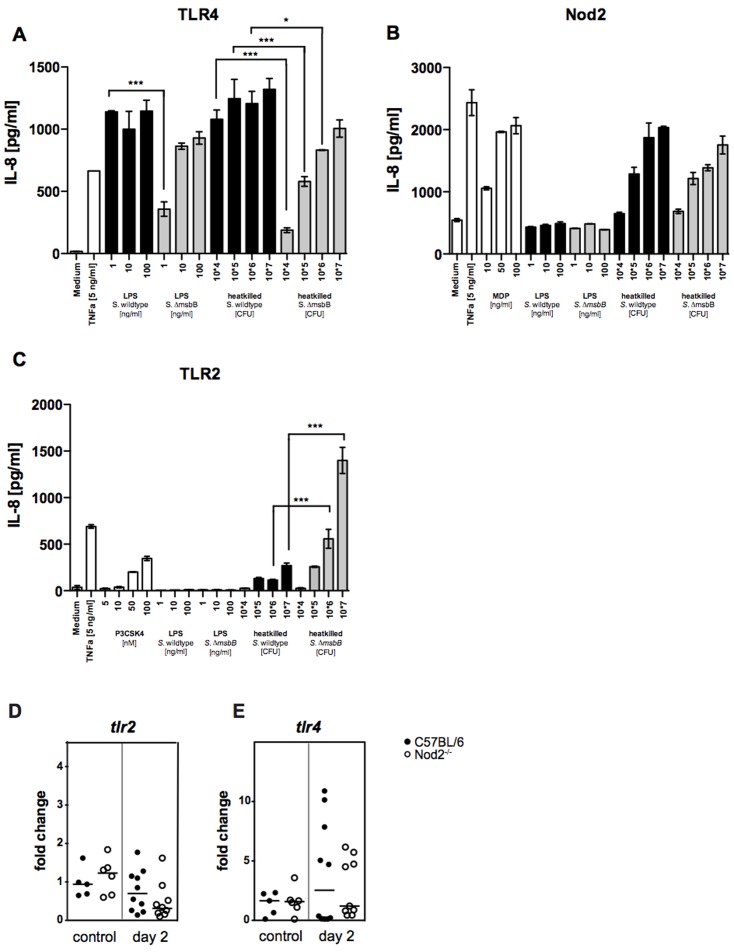
Reduced TLR4, but enhanced TLR2 reactivity of *S.* Typhimurium Δ*msbB*. HEK293 cells were transiently transfected with (A) TLR4, (B) Nod2 or (C) TLR2 and stimulated with purified LPS either of *S.* Typhimurium wild-type or *S.* Typhimurium Δ*msbB* or with heat-killed bacteria. As a negative control only medium was added. Positive controls used were muramyldipeptide (MDP) for Nod2 stimulation and the synthetic lipopeptide *N*-palmitoyl-*S*-[2,3-bis(palmitoyloxy)-(2R,*S*)-propyl]-(R)-cysteinyl-seryl-(lysyl)3-lysine (P_3_CSK_4_) for TLR2 stimulation. IL-8 release was measured by ELISA. Shown are representative experiments of 3 independent experiments. Statistical analysis: 1way ANOVA with Bonferroni's multiple comparison post-test. * *p*<0.05; *** *p*<0.001; only significant differences between the same concentrations of wild-type and Δ*msbB* are marked. D–E: Gene expression of *tlr2* and *tlr4* in the cecum of mock infected or *S.* Typhimurium Δ*msbB* infected mice 2 days post infection was measured by quantitative real-time PCR. Data were normalized to *gapdh* expression levels; Statistical analysis: 1way ANOVA with Tukey's multiple comparison post-test. No significant differences were observed.

To investigate which PRRs are involved in the *S.* Typhimurium Δ*msbB* mutant's ability to trigger exacerbated inflammation in *Nod2^−/−^* mice, we exploited HEK293 cells, which do not express most PRRs. HEK293 cells were transfected with various PRRs and stimulated with either wild-type or Δ*msbB* LPS. Subsequently, IL-8 production was measured by ELISA as a downstream indicator that a specific PRR was stimulated. Firstly, HEK293 cells were transfected with human TLR4 (together with human CD14 and MD2) and stimulated with purified LPS from wild-type *S.* Typhimurium or from the Δ*msbB* mutant or with heat-killed wild-type *S.* Typhimurium or with heat-killed Δ*msbB* mutant bacteria. Upon stimulation with wild-type LPS, TLR4-transfected HEK293 cells produced high amounts of IL-8 (Figure 4A). When cells were stimulated with low concentrations of LPS from *S.* Typhimurium Δ*msbB*, significantly less IL-8 was produced. No significant differences were observed using high concentrations of LPS. Similarly, stimulation with wild-type *Salmonella* induced higher IL-8 levels than stimulation with *S.* Typhimurium Δ*msbB* (Figure 4A). These data corroborate previously published data [17] that showed that *S.* Typhimurium Δ*msbB* LPS has a diminished ability to activate TLR4. Stimulation of Nod2-transfected HEK293 cells with LPS isolated from wild-type *S.* Typhimurium and the Δ*msbB* mutant or with the wild-type and mutant bacteria resulted in no significant differences in the amount of produced IL-8 (Figure 4B). In addition, in TLR2-transfected HEK293 cells, as expected, no IL-8 was produced upon stimulation with purified LPS from either wild-type or mutant bacteria (Figure 4C). In contrast, stimulation of TLR2-transfected HEK293 cells with heat-killed bacteria resulted in drastically increased IL-8-production after stimulation with *S.* Typhimurium Δ*msbB* compared to wild-type bacteria (Figure 4C). Next, we tested if in vivo bacterial infection altered expression of TLRs. We did not see any significant changes in expression of *tlr2* or *tlr4* two days post infection with *S.* Typhimurium Δ*msbB* (Figure 4D–E). These results suggest that *S.* Typhimurium Δ*msbB* triggers excessive pro-inflammatory cytokine production through enhanced stimulation of TLR2.

### Nod1 contributes to *S.* Typhimurium Δ*msbB*-induced colitis

In line with our results using wild-type *S.* Typhimurium C5, Geddes *et al.* recently reported that oral infection with wild-type *S.* Typhimurium SL1344 results in similar cecal inflammation in C57Bl/6, *Nod1^−/−^* or *Nod2^−/−^* mice [5]. However, mice deficient in both Nod1 and Nod2 develop less inflammation and less pro-inflammatory cytokine production but have increased *Salmonella* colonization. Consequently, we next wanted to address whether increased *S.* Typhimurium Δ*msbB*-triggered inflammation is solely dependent on Nod2 or whether Nod1 also plays an important role. Accordingly, C57Bl/6, *Nod1^−/−^* and DKO mice were infected with *S.* Typhimurium Δ*msbB*.

**Figure 5:**
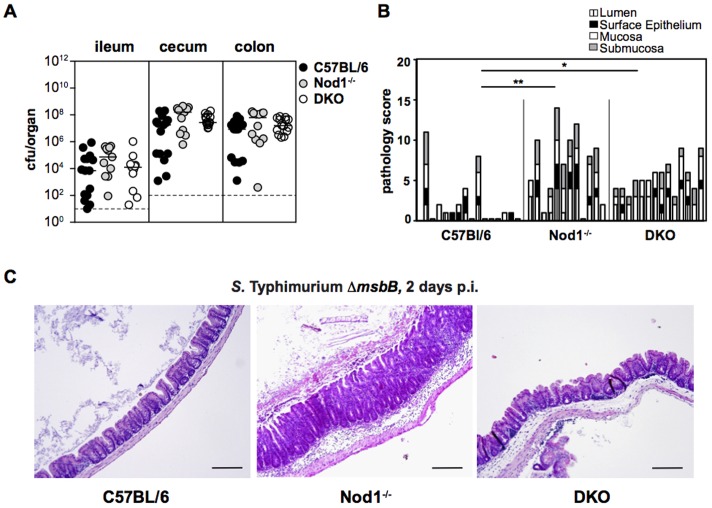
Nod1 also dampens *S.* Typhimurium Δ*msbB*-induced colitis. Streptomycin-pretreated C57Bl/6, *Nod1^−/−^* and DKO mice were orally infected with *S.* Typhimurium Δ*msbB* for 2 days. (A) *S.* Typhimurium colonization of ileum, cecum and colon. Dashed line: limit of detection; cfu: colony forming units. (B) Pathology scores of infected ceca. Statistical analysis: 1way ANOVA with Bonferroni's multiple comparison post-test. * *p*<0.05; ** *p*<0.01. (C) H&E staining of infected cecum sections. Original magnification 200×; scale bars = 100 µm.

At day 2 p.i., C57Bl/6, *Nod1^−/−^* and DKO mice were colonized with comparable levels of *S.* Typhimurium Δ*msbB* (Figure 5A). However, inflammation was significantly more pronounced in *Nod1^−/−^* and DKO mice compared to C57Bl/6 mice (Figure 5B). More specifically, more extensive submucosal edema was present in *Nod1^−/−^* and DKO mice as compared to C57Bl/6 mice. Additionally, exacerbated inflammation in *Nod1^−/−^* and DKO mice was characterized by increased inflammatory infiltrates, apoptotic epithelial cells in the lumen and initiation of the destruction of the crypt structure. Overall, exacerbated inflammation in *Nod1^−/−^* and DKO mice indicates that both Nod2 and Nod1 function are important for control of early *S.* Typhimurium Δ*msbB*-triggered cecal inflammation.

## Discussion

Pattern recognition receptors are crucial for immune homeostasis in the gut and during infection with pathogens. TLRs and NLRs recognize distinct microbial structures and their activation leads to the production of pro-inflammatory cytokines and chemokines and to the recruitment of immune cells to the site of infection. Mutations in PRRs are linked not only to susceptibility to various infectious diseases but also to inflammatory bowel diseases such as CD and ulcerative colitis (reviewed in [6]). For example, mutations in Nod2 are major risk factors for developing CD in the Caucasian population [7] [8]. How a non-functional Nod2 protein can lead to chronic uncontrolled inflammation is still not completely understood.

*S.* Typhimurium Δ*msbB* has been considered as a potential anti-cancer vaccine strain [23] [24]. This mutant is missing an acyl residue on its LPS and thus has diminished TLR4 reactivity and decreased virulence *in vivo*. Here, we demonstrate how the *msbB* mutation affects Nod1- and Nod2-mediated intestinal inflammation. We observed delayed clearance of *S.* Typhimurium Δ*msbB* in *Nod2* ^−/−^ mice. In addition, we detected increased inflammation in the cecum of *Nod2* ^−/−^, *Nod1* ^−/−^ and DKO mice.

Nod2 has been shown to be critical for the defense against various other pathogens such as *Listeria monocytogenes, Citrobacter rodentium*, *Helicobacter hepaticus* and *Mycobacterium tuberculosis* [21] [25] [26] [27]. In particular, *Citrobacter* induced less MCP-1/CCL2 and persisted longer in *Nod2* ^−/−^ mice compared to wild-type mice [26]. Similarly, we also observed a delayed clearance of *S.* Typhimurium Δ*msbB* in *Nod2* ^−/−^ mice and this was associated with higher numbers of neutrophils in the gut lumen. A recent study showed that *Salmonella* can reside in luminal neutrophils for a short time [28]. Therefore, one could postulate that these luminal neutrophils in *Nod2* ^−/−^ mice may harbor *Salmonella* Δ*msbB* and thereby facilitate extended persistence despite the elevated early inflammation in these mice.

Colonization and inflammation induced by infection with wild-type *S.* Typhimurium bacteria in Nod1- or Nod2-deficient mice was similar to C57Bl/6 mice, which is in agreement with a previously published report [5]. Furthermore, wild-type *Salmonella* triggered significantly milder cecal inflammation in DKO mice compared to C57Bl/6 mice as a result of less pro-inflammatory signaling [5]. These data suggest that in the absence of Nod1 or Nod2, each NLR can compensate for the other. *Geddes et al.* also demonstrated that early *Salmonella*-induced inflammation is in part triggered by innate Th17 cells [29]. Using infection with the *S.* Typhimurium Δ*msbB* mutant, we could not detect upregulation of IL-17 expression at day 2 p.i. (not shown). This may be due to the delayed and overall lower inflammation at this time point by the attenuated Δ*msbB* mutant compared to the inflammation triggered by wild-type *Salmonella*.

In this current work, we demonstrate that the *S.* Typhimurium Δ*msbB* mutant is able to trigger enhanced inflammation when either Nod1, Nod2, or both Nod1 and Nod2 are absent. This is in stark contrast to the results obtained by infection of these knockout mice with wild-type *Salmonella* and could be due to synergistic or antagonistic crosstalk between NLRs and TLRs. Our *in vitro* data show that *S.* Typhimurium Δ*msbB* triggers decreased TLR4-dependent, but highly increased TLR2-dependent pro-inflammatory signaling. This could be due to better accessibility or higher expression of lipoproteins (i.e. TLR2 ligands) on the bacterial surface [30]. However, enhanced TLR2 signaling should cause similar inflammation in C57Bl/6 and *Nod2* ^−/−^ mice. A possible reason for the increased pathology in *Nod2* ^−/−^ mice could be that the lack of negative regulatory signals from Nod2 on TLR2 signaling leads to enhanced pro-inflammatory cytokine secretion and inflammation in the gut. A negative regulatory role of Nod2 on TLR responses has indeed recently been demonstrated. For example, Nod2 dampens TLR2-mediated inflammation in a model of T cell-dependent colitis [31]. Similarly, Nod2 stimulation reduces LPS-triggered TLR4 activation [32] and is protective in a model of LPS-induced necrotizing enterocolitis [12]. And lastly, silencing of Nod2 in RAW macrophages results in enhanced NF-κB expression demonstrating that in the absence of stimulation, Nod2 might have inhibitory effects on TLR4 signaling [13].

On the other hand, synergistic effects between TLR and NLR signaling have also been demonstrated. For instance, stimulation of Nod1 or Nod2 can lead to increased TLR4 signaling [33] [34]. Nod2 stimulation was also able to synergistically enhance TLR4-, TLR2- and TLR3-dependent cytokine production [35]. More detailed analyses demonstrated that stimulation of Nod2 with low doses of MDP enhanced TLR2 responses while stimulation with high doses of MDP inhibited TLR2 responses [36] [10]. Importantly, synergistic effects between NLRs and TLRs have primarily been shown in settings of acute activation. In contrast, in the gut, where NLRs and TLRs are chronically exposed to their ligands, chronic stimulation of Nod2 leads to tolerance towards TLR2- and TLR4-induced pro-inflammatory cytokine production [14] [37]. Downregulation of cytokine expression by chronic Nod2 stimulation could be either due to downregulation of PRR expression (e.g. TLR2) or induction of anti-inflammatory mediators or decoy receptors (such as SIGIRR) [38]. In contrast, Barreau *et al.* showed that TLR2 and TLR4 were upregulated in Nod2 deficient mice under steady state conditions [9] which may lead to a further enhanced TLR2/TLR4-mediated pro-inflammatory response.

In addition to *Nod2* ^−/−^ mice, we show that the *S.* Typhimurium Δ*msbB*-mutant causes also more inflammation in *Nod1* ^−/−^ and DKO mice compared to C57Bl/6 mice indicating that both NLRs are capable of dampening the inflammatory response to *S.* Typhimurium. While Nod2 has been shown to be an important risk factor for IBD, there are contradictory data about the role of Nod1 in IBD [39]. Some studies showed that mutations in Nod1 predispose individuals to IBD [40] whereas others could not find any association [41]. However, it seems clear that Nod1-deficient mice are more susceptible to bacterial infections such as *Clostridium difficile* [42] and Nod1 is important for the interaction of *S.* Typhimurium with dendritic cells [43].

In conclusion, we demonstrate that Nod1 and Nod2 dampen early intestinal inflammation triggered by *Salmonella* Δ*msbB*, mainly via TLR2. As *Salmonella* Δ*msbB* is under investigation as an anti-cancer vaccine strain, our results indicate that this strain may cause inflammatory complications at least in persons with mutations in Nod1 or Nod2 pathways.

## Supporting Information

Figure S1 **Similar colonization of C57Bl/6 and** ***Nod2^−/−^*** **mice infected with wild-type** ***S.*** **Typhimurium.** (A) Streptomycin-pretreated C57Bl/6 and *Nod2^−/−^* mice were orally infected with wild-type *S.* Typhimurium SL1344 for two days. Colonization of intestinal organs is shown. No significant differences in colonization were observed. The dashed line indicates the limit of detection. cfu: colony forming units. Statistical analysis one-way ANOVA with Tukey's multiple comparison post-test after logarithmic transformation. (B) Pathology score of *S.* Typhimurium Δ *msbB* infected C57Bl/6 and *Nod2^−/−^* mice at day 7 post infection showing no significant differences. Statistical analysis: Student's t-test.
(TIFF)
