# Supplementary figures and images for: Salmonella enterica serovar Typhimurium ΔmsbB Triggers Exacerbated Inflammation in Nod2 Deficient Mice
Source: PLoS One. 2014 Nov 25;9(11):e113645. doi: 10.1371/journal.pone.0113645 (PMC4244092; doi:10.1371/journal.pone.0113645)

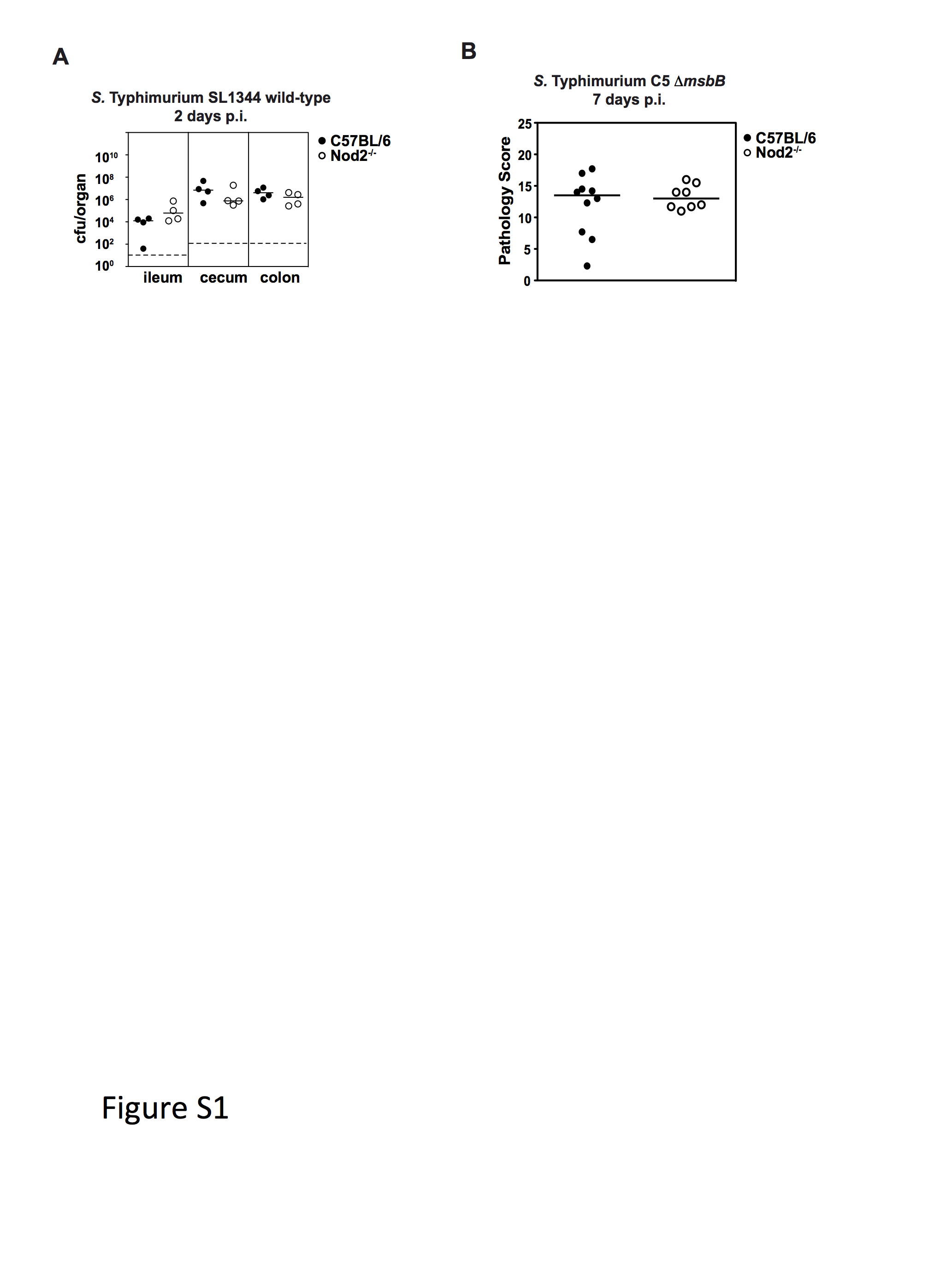

Supplement: Figure S1 — Similar colonization of C57Bl/6 and Nod2−/− mice infected with wild-type S. Typhimurium. (A) Streptomycin-pretreated C57Bl/6 and Nod2−/− mice were orally infected with wild-type S. Typhimurium SL1344 for two days. Colonization of intestinal organs is shown. No significant differences in colonization were observed. The dashed line indicates the limit of detection. cfu: colony forming units. Statistical analysis one-way ANOVA with Tukey's multiple comparison post-test after logarithmic transformation. (B) Pathology score of S. Typhimurium Δ msbB infected C57Bl/6 and Nod2−/− mice at day 7 post infection showing no significant differences. Statistical analysis: Student's t-test. (TIFF) [file pone.0113645.s001.tiff]
